# Supplementary material for: An integrated photonic engine for programmable atomic control
Source: Nat Commun. 2025 Jan 2;16:82. doi: 10.1038/s41467-024-55423-3 (PMC11696560; doi:10.1038/s41467-024-55423-3)
Supplement: Supplementary file 1 — Supplementary Information [file 41467_2024_55423_MOESM1_ESM.pdf]

Supplementary Information: An integrated photonic engine for programmable atomic control

Ian Christen[1,*], Thomas Propson[1], Madison Sutula[1], Hamed Sattari[2], Gregory Choong[2], Christopher Panuski[1], Alexander Melville[3], Justin Mallek[3], Cole Brabec[1], Scott Hamilton[3], P. Benjamin Dixon[3], Adrian J. Menssen[1], Danielle Braje[3], Amir H. Ghadimi[2], and Dirk Englund[1,*]

[1] *Research Laboratory of Electronics, Massachusetts Institute of Technology, Cambridge, MA 02139, USA*
[2] *Centre Suisse d'Electronique et de Microtechnique (CSEM), 2000 Neuchâtel, Switzerland*
[3] *Lincoln Laboratory, Massachusetts Institute of Technology, Lexington, MA 02421, USA*

Keywords: photonic integrated circuits, lithium niobate on insulator, thin-film lithium niobate, large-scale, multi-channel, visible modulator, silicon-vacancy, quantum control

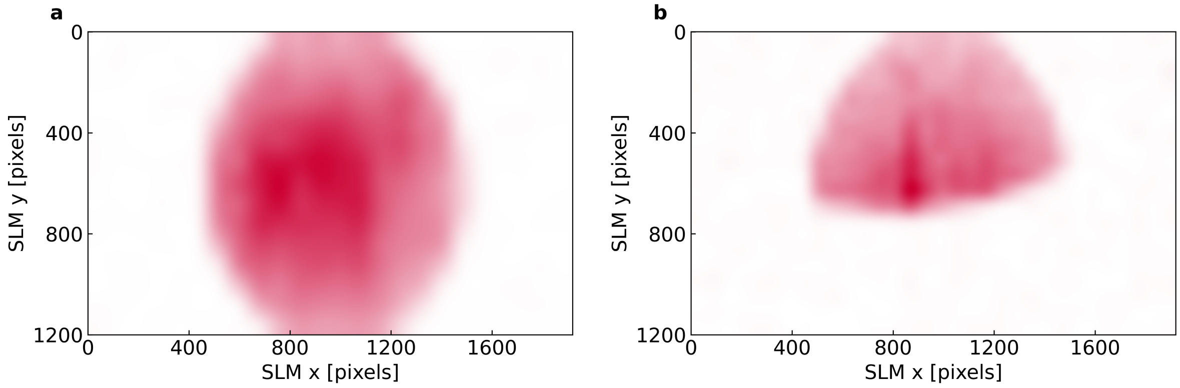

FIG. S1: **Fanout SLM amplitude distributions.** The measured source amplitude distributions on the fanout SLM **a**, for 780 nm and **b**, for 737 nm.

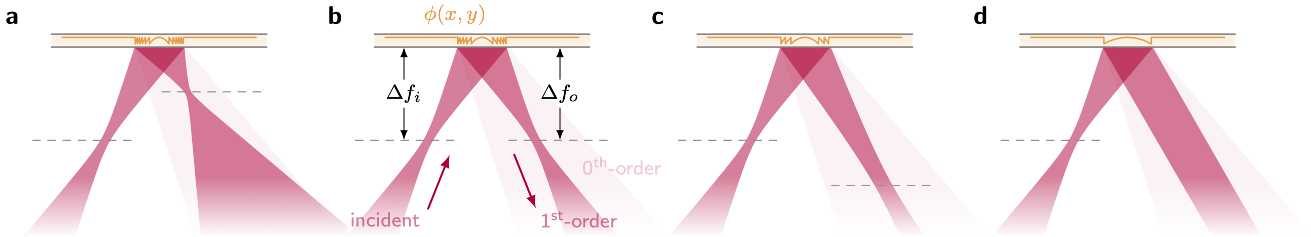

FIG. S2: **Microlens fill factor conversion.** The influence of a variety of Δf_o upon the fill factor η_o of the output beam: **a**, $\Delta f_o = .5\Delta f_i$, **b**, $\Delta f_o = \Delta f_i$, **c**, $\Delta f_o = 1.5\Delta f_i$, and **d**, collimated output.

*Electronic address: ichr@mit.edu, englund@mit.edu

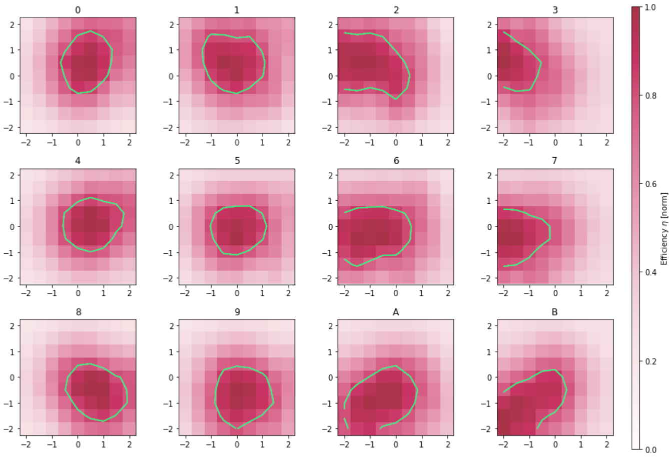

FIG. S3: **Microlens steering range.** Lens efficiency η versus normalized steering range R for twelve channels with $\Delta f_o \approx \Delta f_i$. The x and y axes are normalized to the channel pitch Γ. Contours denote 80% efficiency.

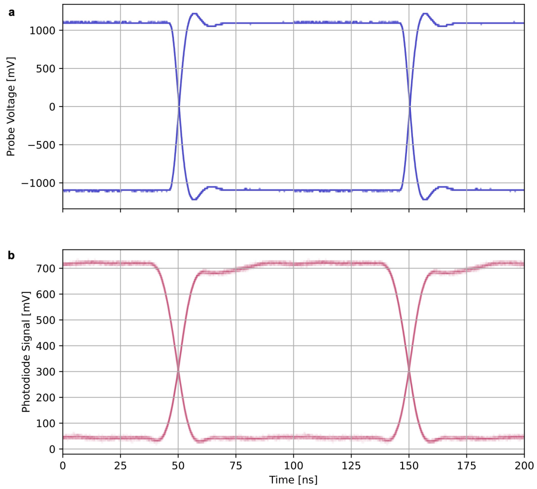

FIG. S4: **Time domain modulator performance. a,** An input voltage in the form of a square wave (sourced from 70 MHz bandwidth AWG) is imparted to a modulator channel, imparting the signal to **b,** the optical domain, detected by a 50 MHz bandwidth photodiode.

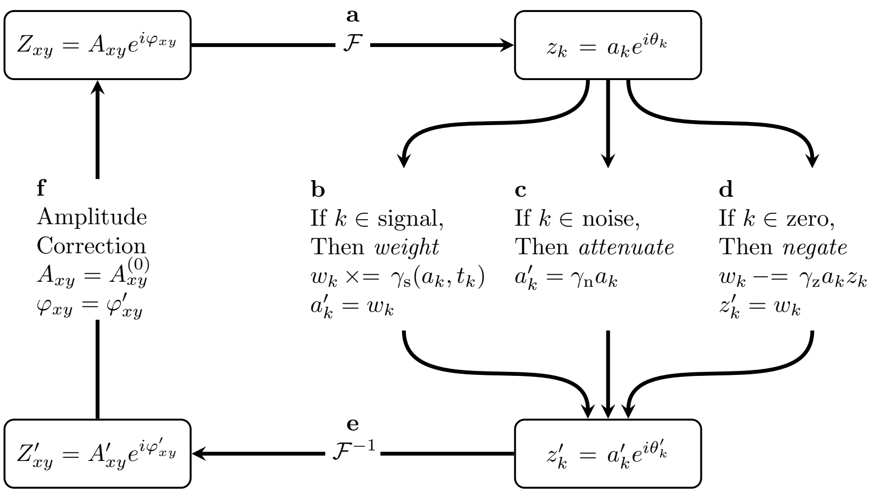

FIG. S5: **Modified WGS loop.** Weighted Gerchberg-Saxton (WGS) numerically solves the problem of phase retrieval: what nearfield phase pattern φ_{xy} best approximates a desired farfield amplitude distribution $a_k^{(0)}$? **a**,**e,** The loop consists of iterations transforming from nearfield to farfield and back. Here, $\mathcal{F}$ represents either a discrete Fourier transform of the 2D nearfield xy space resulting in a 2D grid of farfield points k, or a pointwise transformation of the nearfield phase to desired k points via a kernel specific to each specific k. Each iteration is accompanied by corrections to the nearfield or farfield based on known quantities: **b**, WGS balances power between spots to converge on the distribution $a_k^{(0)}$ while preserving the current estimated phase θ_k and **c**, MRAF adds a term allowing power to remain in a noise region to better match $a_k^{(0)}$ in the signal region. **d**, We additionally explore an additive destructive interference correction to negate power at a spot. **f**, The nearfield correction fixes the nearfield amplitude to measured values, completing the loop.

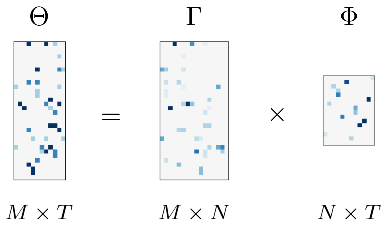

FIG. S6: **Quantum Circuit Factorization.** Gate rotations are represented as real matrix Θ (color corresponds to the strength of a matrix element. In some cases, this matrix can be factored into two others representing the state of fast modulators Φ and the configuration of the fanout Γ. The case shown here uses $(M, N, T) = (32, 16, 12)$.

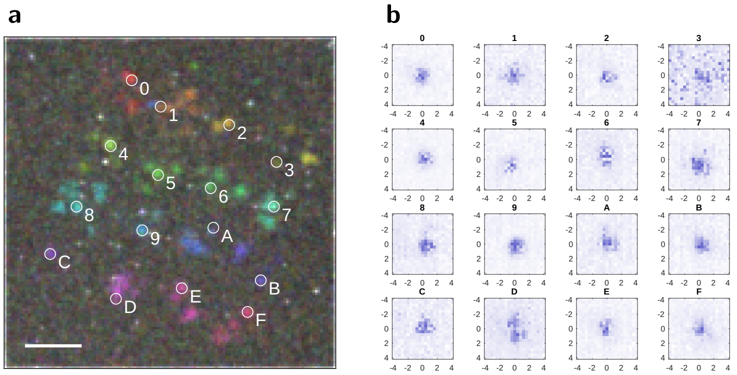

FIG. S7: **Channel-emitter alignment.** **a**, Composite blaze scans from each microlens (colored by hue) are used to select an isolated emitter at a target camera pixel for each channel (0-F). Scalebar represents 5 μm. **b**, After optimization in x, y, and focus, blaze scans collecting on the target pixel show centered fluorescence. The x and y axes use units of blaze angle at the beamsteering SLM in milliradians.

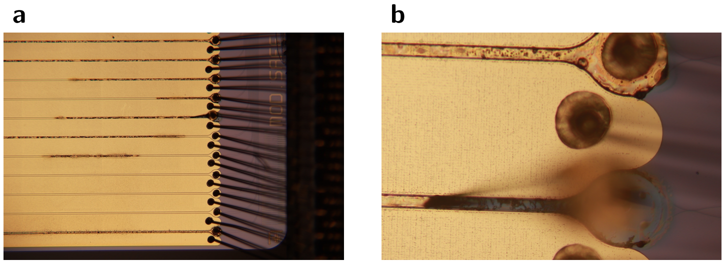

FIG. S8: **Electrical breakdown damage.** **a**, Overview and **b**, zoom upon modulator charring and delamination which we attribute to an AWG over-voltage state.

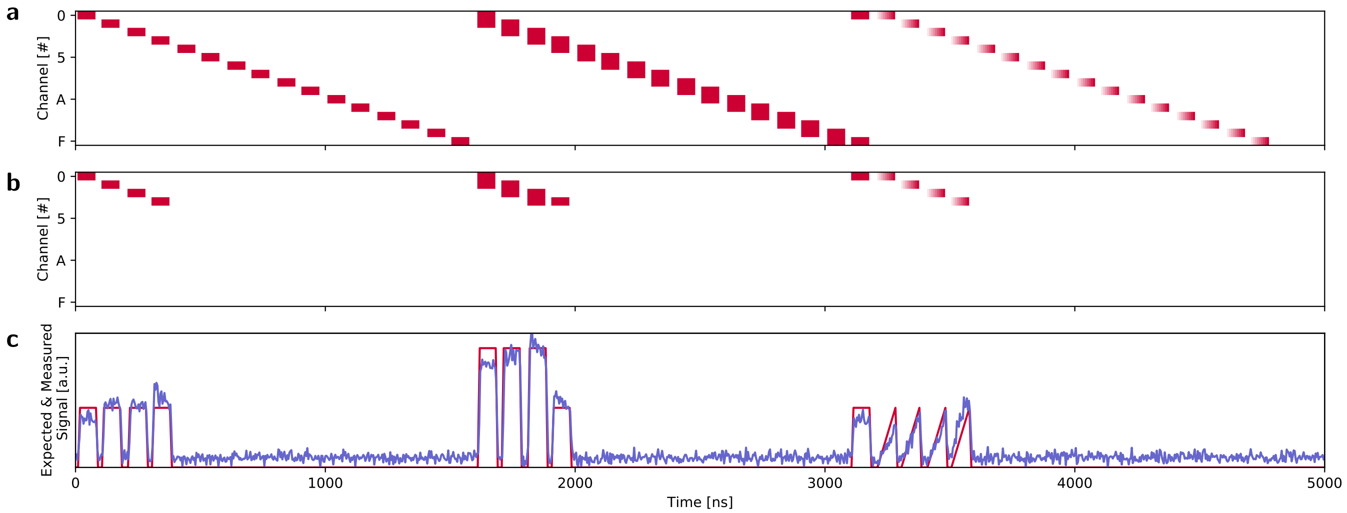

FIG. S9: **Spatial Addressing Data.** **a**, Target pulse sequence on sixteen channels. Color represents channel amplitude for a given channel at a given time. **b**, Pulse sequence with our truncated channel count. **c**, Expected signal (red) as the sum of all globally-collected channels, compared with the measured fluorescence (blue).
